# Editorial: New pathways in retirement research: Innovative perspectives on social inequalities and the distribution of transitional risks

**DOI:** 10.3389/fsoc.2022.984874

**Published:** 2022-09-14

**Authors:** Anna Wanka, Moritz Hess, David Lain

**Affiliations:** ^1^Linking Ages, Goethe University Frankfurt, Frankfurt, Germany; ^2^Hochschule Niederrhein University of Applied Sciences, Mönchengladbach, Germany; ^3^Newcastle University, Newcastle upon Tyne, United Kingdom

**Keywords:** retirement, social inequalities, transition, flexibility, risk

The transition from work to retirement remains one of the major turning points within an individual's life course, as it entails a variety of significant changes. Income and occupational prestige may decrease, but so does work-related stress. Similarly, work-related social networks may loosen, but private networks can be strengthened, and time is freed up for formerly neglected or new tasks and activities (Atchley, 1975). Studies have explored why people retire (individual motivations), how they retire (institutional pathways), and how retirement affects different dimensions of older adults' lives, including their health, wellbeing, finances, social networks, and activities (Henkens et al., 2018).

We know that individuals retire in different ways and experience their retirement differently, but beyond that, research also strongly suggests that the retirement transition is closely related to markers of social inequalities (Hofäcker et al., 2015). How a person experiences retirement both as a transition and as a life stage is likely to be influenced by the nature of their (previous) employment and by their gender, marital status, ethnicity, and social class. Retirement transitions may also be influenced by welfare legislation in a particular country and by discourses and norms around “right” retirement ages, and inequalities may be enhanced or even altered to some degree by policies to extend working lives (Hofäcker et al., 2015). Such policy changes include increases in pension ages, the closure of early retirement options, the lowering of replacement rates, and, in some countries, the abolition of mandatory retirement ages (Harper, 2015).

Against the context of demographic aging, policies increasingly aim at delaying retirement in an attempt to “extend working lives.” This aim is implemented through, for example, “activating” older employees and keeping them in the labor market as long as possible, while at the same time framing their (in-)ability to do so in terms of individual responsibility (Lain et al., 2022). However, not all older workers are able to keep working until a rather old age. Concerns are, hence, raised that social inequalities in retirement transitions are increasing (Hofäcker et al., 2015). At the same time, research suggests that ageism is still widespread among employers, resulting in older employees being viewed as less productive, less likely to be invested in and more likely to be offered early retirement routes (Stypińska and Nikander, 2018). This results in a new and pronounced form of “structural lag” between societal expectations (and, often, individual preferences) on the one hand and institutionalized stereotypes and limited possibilities to work for older adults on the other. In this context, pressures to extend working lives may increase inequalities among those working longer and among those in retirement.

Thus, social inequalities and the distribution of transitional risks are in the focus of research. Understanding and explaining these inequalities as well as their extent, roots and consequence is not only relevant from an academic perspective but is of utmost importance from a societal and practical perspective. Against this backdrop, this Special Issue Topic focuses on social inequalities regarding retirement. It consists of eight studies authored by researchers from the Netherlands, Belgium, Germany, the United Kingdom, Austria, Italy, Sweden, and Poland, all revolving around inequalities in retirement transitions, but doing so from different perspectives and using a variety of qualitative and quantitative methodological approaches. Addressed topics are inequalities in (planned) retirement timing, ageism, retirement practices and meanings, resonance of retirement transitions, flexible retirement as well as life-expectancy and healthcare usage in retirement.

In the context of financial pressures to extend lives, the paper by Hess et al. investigates the extent to which individuals are adapting their retirement expectations. They do this by analyzing the planned retirement ages of older workers in Europe using data from the Survey of Health, Ageing and Retirement in Europe (SHARE). Their analysis suggest that people's plans are adapting: across the 10 European countries investigated there is an increase in planned retirement age of 1.36 years.

While people's expectations about the retirement timing might be changing, we might expect increases in employment to be unevenly spread across different segments of the population. De Luigi et al. examine the planned retirement timing of Italian women using the Labour Force Survey and a Heckman selection model. They find that the comparably high planned retirement ages of women in Italy are mainly driven by those with low and medium education. The authors explain that this group often has fragmented employment histories and therefore faces financial pressures to postpone retirement.

Issues of fairness related to extended working lives are also addressed in the paper by Deeg et al. One of the central equity issues in pensions policy relates to life expectancy - the potential period of time for which an individual can expect to benefit from receiving a pension. The authors explore occupation-based differences in life expectancy using data from the Dutch population-based Longitudinal Aging Study Amsterdam (LASA). They find that those working in non-skilled general, technical, and transport have shorter life expectancies than those in professional roles. It is argued that these occupation-based differences in life expectancy contort the actuarial fairness of pension system, as those with higher expectancy will receive benefits for a longer time. As a result, they argue that pension ages should be adapted to life expectancy projections for different occupational groups.

Scherger's conceptual paper also provides a critical lens on policy to extend working lives. In policy circles, it is often assumed that “flexibilizing the retirement transition” is an innovative and humane way of extending working lives. While flexible work is seen as helping people to work longer, “flexibility” is an ambiguous term that can have both positive and negative outcomes in different circumstances. Scherger therefore develops a conceptual framework to help future research make sense of these different “dimensions” of flexibility. As she makes clear, access to “flexible retirement” is unequally distributed if people, for example, are financially unable to reduce their hours. Likewise, “flexibilization” can also be understood in terms of shifting responsibility onto individuals to take responsibility for their extended working lives. Scherger's paper therefore makes an important contribution, enabling researchers to analytically account for the seemingly contradictory dimensions within the flexibilization in later life.

As with the previous paper discussed, Vickerstaff and Van der Horst also interrogate a topic central to debates about older workers and extended working lives: age norms and stereotypes. Commonly, previous research has conceptualized this in relation to stereotypes held by managers toward older workers, and the implications of this for their treatment at work. In this paper the authors suggest that such as focus on ageism, while important, is incomplete. They argue that older individuals are also likely to internalize negative norms of what it means to be older in social contexts such as the workplace. To examine this, they analyse semi-structured qualitative interviews with employees and managers in the United Kingdom. A “decline narrative” of aging is shown to be widespread among both groups, but this can have different effects on retirement planning—from serving as a motivation to retire early, or, conversely, stay employed longer.

The papers by Urbaniak and Bischoff et al. also draw on qualitative research to examine the lived experience of retiring. Urbaniak's paper draws on a qualitative, practice-theoretical approach to explore how social inequalities are (re-)produced in everyday retirement practices and meanings in Poland. She finds four broad types of retirement practices—caregiving, working, exploring and disengaging—and discusses how older adults redefine the meanings of the term “retiree” in the Post-Communist context. These practices and meanings, she argues, reflect structural and individual inequalities at the intersection of gender, age, and socioeconomic status.

Equally drawing on practice theories, Bischoff et al. deploy a mixed-methods research design to explore the affective transitional experiences and retirement practices of older adults in Germany. Their paper explores how relations between the self and the world transform when people retire, which social practices facilitate this process, and which role dimensions of social inequality—such as gender, income, education, or mental health status—play for resonance transformations in this transition. They find that the transition from work to retirement entails a specific “resonance choreography” that differs by retirement pathway, and that people retiring “too early” (based on chrono-norms) or on atypical pathways (e.g., unemployment) tend to experience more dissonance in the retiring process, which can in turn affect their wellbeing and health.

The final paper in this Research Topic explores another topic of interest to aging societies and retirement: healthcare usage. This topic is explored in the paper by Wetzel et al. The authors examine how secondary healthcare usage changes during the retirement transition. Analysing Swedish register data, they find no overall changes in secondary healthcare use with retirement, but differences based on gender and education. Following the retirement transition, gender differences in secondary healthcare use are shown to decrease, while within-gender educational differences tend to increase. These changes, they argue, can affect health and life expectancy in the long run, and reflect inequalities explored elsewhere in this Research Topic.

Taken as a whole, this Research Topic of papers makes an important contribution to debates on employment and retirement in older age. It covers a range of European countries, and empirically examines issues of importance to policy including retirement planning, life expectancy, healthcare usage, and inequalities in employment. Conceptually and empirically it also contributes to our understandings of the flexibilization of retirement, internalized age norms, and the lived experience and meanings of “retirement.” It offers new and innovative perspectives on inequalities before, in and after the retirement transition.

## Author contributions

All authors listed have made a substantial, direct, and intellectual contribution to the work and approved it for publication.

## Conflict of interest

The authors declare that the research was conducted in the absence of any commercial or financial relationships that could be construed as a potential conflict of interest.

## Publisher's note

All claims expressed in this article are solely those of the authors and do not necessarily represent those of their affiliated organizations, or those of the publisher, the editors and the reviewers. Any product that may be evaluated in this article, or claim that may be made by its manufacturer, is not guaranteed or endorsed by the publisher.
